# Synthesis and characterization of soluble pyridinium-containing copolyimides†

**DOI:** 10.1039/d4ra06443g

**Published:** 2024-11-21

**Authors:** Anastasiia Hubina, Alina Madalina Darabut, Yevheniia Lobko, Jaromir Hnat, Jan Merna, Karel Bouzek

**Affiliations:** a University of Chemistry and Technology (UCT) Prague Technicka, 5 Prague 166 28 Czech Republic hubina@vscht.cz; b Charles University Prague 180 00 Czech Republic

## Abstract

Novel ionene-type cationic copolyimides based on 4,4′-oxydiphthalic anhydride (ODPA), 4,4′-(1,4-phenylenediisopropylidene)bisaniline (BIS P), and 2,6-diaminopyridine (DAP) were synthesized. The copolyimides were obtained in two stages: first, the copolyimides with the 0/1, 0.2/0.8, 0.3/0.7, 0.5/0.5, 0.6/0.4 and 1/0 DAP/Bis P ratios were obtained through thermal imidization, and then quaternization of soluble copolyimides with methyl iodide was conducted for 24 or 48 h. The samples were characterized *via* FTIR, NMR and EDX methods to confirm their structure and composition. The cationic copolyimides with a DAP content of less than 0.3 showed initial weight loss (onset) at about 250 °C, according to TGA results and demonstrated solubility in chloroform. The highest ionic conductivity value of 0.234 S cm^−1^ was showed by the sample with 0.3 DAP content and 0.15 degree of quaternization. The stability of the membranes in alkaline media was evaluated using FTIR and TGA. It was shown that samples with a DAP content of more than 0.3 lost their integrity probably owing to partial hydrolysis of imide rings, while copolyimides with a DAP content of 0.2 and 0.3 remained stable.

## Abstract 2

Dense pyridinium-containing copolyimide membranes with uniform charge distribution.

## Graphical abstract

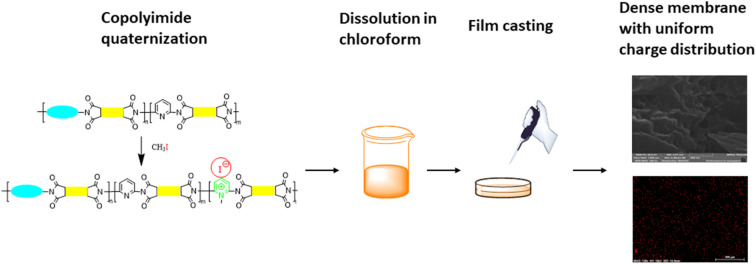

## Introduction

Hydrogen (H_2_) is considered a promising energy storage carrier for industrial applications, transportation and household application. Electrochemical water splitting stands out as a well-established method for generating pure H_2_.^1–4^ Among various water electrolysis (WE) technologies, anion exchange membrane water electrolysis (AEM WE) holds particular significance, although it remains mainly a laboratory technology compared to alkaline water electrolysis (AWE) and proton exchange membrane water electrolysis (PEM WE). Over the past two decades, research interest in advancing AEM WE technology has significantly increased.^5–7^

An anion exchange membrane (AEM) represents an important part of AEM WE. To be effective, an AEM must meet specific criteria: (i) it should facilitate the efficient separation of hydrogen and oxygen produced at the electrodes, (ii) possess high ion-exchange capacity, (iii) exhibit non-permeability to electrons, and (iv) demonstrate robust mechanical stability and (v) ionic conductivity greater than 0.1 S cm^−1^, along with (vi) resistance to degradation in alkaline media.^8–10^

Polymer AEMs typically consist of a polymer backbone, which are responsible for key properties such as mechanical strength, thermal resistance, swelling behaviour, and water absorption, along with cations attached as pendant groups to the main polymer chain, contributing to ion conductivity. Considering that degradation in alkaline environments can result from both polymer chain breakdown and cation destruction, selecting a polymer with an alkali-resistant backbone is crucial.^11,12^

Among the polymers commonly employed in AEMs, poly(arylene ether) and poly(phenylene) are frequently utilized, alongside poly(ethylene), while block or random copolymers such as polystyrene-*b*-poly(ethylene/butylene)-*b*-polystyrene also find application.^13–15^ Although methods for the copolymerization of styrene with vinyl benzyl chloride with subsequent functionalization using imidazole derivatives have been reported,^16^ the synthesis of such types of polymers often involves the chloromethylation step, followed by quaternization. However, chloromethylation encounters a significant obstacle due to the carcinogenic properties of conventional chloromethylating agents.^17^ In this regard, one of the more appealing alternatives involves post-polymerization quaternization of polymers containing either tertiary amines or nitrogen-containing heterocyclic fragments in the main chain.

Nevertheless, polymers for AEMs are not only those with cationic functional groups attached to the pendant chain but also those with charges embedded within the polymer backbone.^18^ Recent studies, such as^19^ those dealing with the influence of ion positioning on both ionic conductivity and stability, have unveiled that ionene-type polymers demonstrate higher conductivity and prolonged alkaline stability comparing to ionomers, which bear cation in pendants.^20–22^

Aromatic polyimides (PIs) are renowned for their exceptional thermal stability, resistance to solvents, and mechanical strength.^23–26^ Recently, polyimide-based ionenes gained considerable attention as promising polymers for use as AEMs in various processes, including electrodialysis, fuel cells, and electrolyzers. For instance, in a study,^27^ imidazolium-containing polyimide ionene has been reported, and pyridinium-containing polyimides were developed.^28^ It is well known that pyridinium exhibits lower stability in alkaline media than trimethyl ammonium or imidazolium^29,30^ due to the hydroxide attack on the *ortho* position and the resulting loss of positive charges, which are needed for the transport of hydroxide anions. However, pyridinium-containing polymer AEMs show rather high alkaline stability when the *ortho* position (2,6) of pyridine is occupied, thus preventing the OH^−^ attack.^31–33^

To our knowledge, pyridinium-containing *co*PIs have been synthesized for electrodialysis application but have not been prepared for application in membrane alkaline water electrolysis. This work aims to synthesize soluble polyimide-based ionenes for AEM WE containing pyridinium in the main chain by the postpolymerization quaternization of pyridine-containing *co*PIs. To overcome in general the poor solubility of aromatic PIs in organic solvents, we choose the monomer with methyl substituents, which after introduction into the backbone can enhance the solubility of PI, as shown in a previous report.^34^ To synthesize soluble *co*PI, we initially selected a polyimide based on 4,4′-oxydiphthalic anhydride (ODPA) and 4,4′-(1,4-phenylenediisopropylidene)bisaniline (BIS P), which was reported to possess excellent thermal and mechanical properties and solubility in chloroform^35^ as well as outstanding film-forming ability. In this study, we synthesized a series of copolyimides with different contents of 2,6-diaminopyridine, which were then quaternized to obtain anion exchange membranes. The obtained polyimides were then quaternized and evaluated with respect to their thermal properties, alkaline stability, and ion conductivity.

## Experimental

### Materials

4,4′-Oxydiphthalic anhydride (ODPA) was purchased from Sigma Aldrich and dried in vacuum at 160 °C for 5 h before use. Bisaniline P (BIS P, 4,4′-(1,4-phenylenediisopropylidene)bisaniline) (Mitsui PI), 2,6-diaminopyridine (DAP), methyl iodide, and *N*-methyl-2-pyrrolidone (NMP) were purchased from Merck, while dimethylformamide (DMF) and diethyl ether were purchased from Lachner and used as received.

### Synthesis

**Scheme 1:**
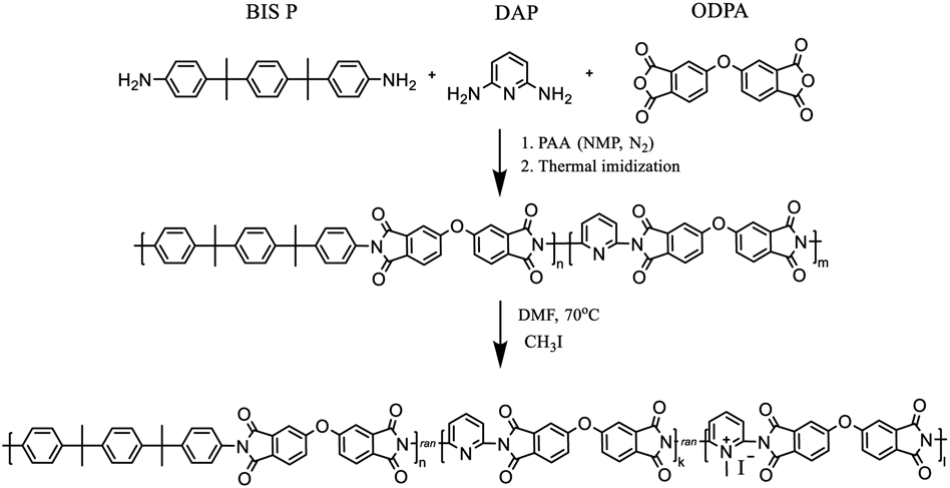
Synthesis of random copolyimides and their modification.

PIs and random *co*PIs were synthesized in two stages (Scheme 1).

First, the dianhydride (ODPA) (2 g, 6.4 mmol) was reacted with either BIS P, DAP or their mixture in NMP under N_2_ atmosphere, which resulted in the formation of the corresponding polyamic acid (PAA) solution in NMP. Samples with the following molar ratio of components were prepared: ODPA : BIS P : DAP as 1 : 1 : 0; 1 : 0, 8 : 0, 2; 1 : 0, 7 : 0, 3; 1 : 0, 5 : 0, 5; 1 : 0, 4 : 0, 6. The resulting 10 w/w% solution was stirred under an inert atmosphere (N_2_) for 12 h. After this period, the PAA was cast on the glass substrate, and during the second stage, thermal imidization was fulfilled according to the procedure: 60 °C – 24 h, 100 °C – 1 h, 150 °C – 1 h, 200 °C – 1 h, 250 °C – 0.5 h. The *co*PIs obtained were DAP-0, DAP-0.2, DAP-0.3, DAP-0.5, DAP-0.6, where 0, 0.2, 0.3, 0.5 and 0.6 correspond to the pyridine content. Soluble *co*PIs (0.5 g) were redissolved in DMF (20 mL) on heating, then excess of methyl iodide (20 eq.) was added. The reaction mixture was refluxed at 70 °C for 24 h or 48 h, then precipitated in diethyl ether, filtered off, washed with diethyl ether, and dried. Different reflux times were used in order to evaluate the influence of the reaction time on the degree of quaternization (DQ). The following samples of modified *co*PIs were synthesized: DAP-0.2Q, DAP-0.3Q, DAP-0.5Q (after 24 h) and DAP-0.2Q2, DAP-0.3Q2 (after 48 h). The membranes were cast on a glass substrate from the solution of the polymer in CHCl_3_.

#### *co*PI

^1^H NMR: (500 MHz; CDCl_3_) 1.75 (12H, s), 7.2 (4H, s), 7.35–7.37 (4H, d), 7.4–7.42 (4H, d), 7.47–7.51 (4H, m), 7.56–7.62 (6H, m), 8.01–8.05 (4H, m), 8.10–8.13 (1H, t).

#### *co*PI-Q

^1^H NMR: (500 MHz; CDCl_3_) 1.75 (12H, s), 3.5 (3H, s), 7.2 (4H, s), 7.35–7.37 (4H, d), 7.4–7.42 (4H, d), 7.47–7.51 (4H, m), 7.56–7.62 (6H, m), 8.01–8.05 (4H, m), 8.10–8.13 (1H, t).

### Characterization

The ^1^H NMR spectra of the polymers were measured on a 500 MHz Bruker Avance 500 spectrometer in CDCl_3_ at 25 °C. The FTIR spectra were recorded with the help of a Nicolet 6700 FTIR-spectrometer (Thermo-Nicolet) in the ATR mode (wavenumber range 4000–600 cm^−1^). DSC analyses were performed on a TA Instruments (Discovery DSC250 auto) calorimeter. The temperature range was from 20 °C to 400 °C, temperature rate was 10 °C min^−1^ and N_2_ flow rate was 50 mL min^−1^. The curves from the second heating run were analyzed. TGA measurements were performed on a TA Instruments TGA550 Auto Advanced with a heating rate of 10 °C min^−1^ in N_2_ atmosphere. Scanning electron microscopy (SEM) images were taken using a MIRA 3 (Tescan GmbH, Czech Republic) microscope operating at 10 keV. The SEM images of the surface and cross-section of the membrane were obtained. The cross-section was prepared by cutting the membrane perpendicularly to its edge with a blade to prevent mechanical deformation. To improve the image quality and reduce the charging effects, a 6 nm layer of platinum was sputtered onto the sample surface using a Kurt-Lesker magnetron sputtering system. Chemical composition mapping analysis was carried out using energy-dispersive X-ray spectroscopy (EDX) with a Bruker XFlash detector mounted directly into the SEM, with electron beam energy of 10 keV. The calculation of the elemental compositions was based on the measurements taken from five scans at five different places of the samples. The solubility of all PIs was tested qualitatively in various solvents (approximately 10 mg/5 mL).

Ion conductivity was evaluated using the electrochemical impedance spectroscopy method. LCR bridge Hameg HM8118 was used to accomplish this task. The frequency of the perturbing signal was in the range of 200 kHz to 20 Hz. The maximal amplitude of the perturbing signal was 50 mV. The resistance of the membrane sample was determined in two-electrode arrangement in the through plane direction. Mercury was used for contacting the surface of the membrane and Pt wires immersed in mercury were used to deliver the perturbing signal. The resistance of the membrane was measured in the OH^−^ form and at laboratory temperature (24 °C). The membrane was transferred to OH^−^ form by storing the sample in 0.1 M NaOH at room temperature for 10 days. The resistance of the membrane was evaluated from the Nyquist diagram as the cross section of the measured points with *x*-axis. The ion conductivity *σ* (S m^−1^) was evaluated following the equation *σ* = *l*/(*R* × *S*), where *l* stands for the thickness of the membrane (*m*), *R* represent the membrane resistance (*Ω*) and *S* is the geometrical surface of the membrane (1.57 × 10^−5^ m).

## Results and discussion

### Synthesis and characterization of (co)polyimides

**Fig. 1:**
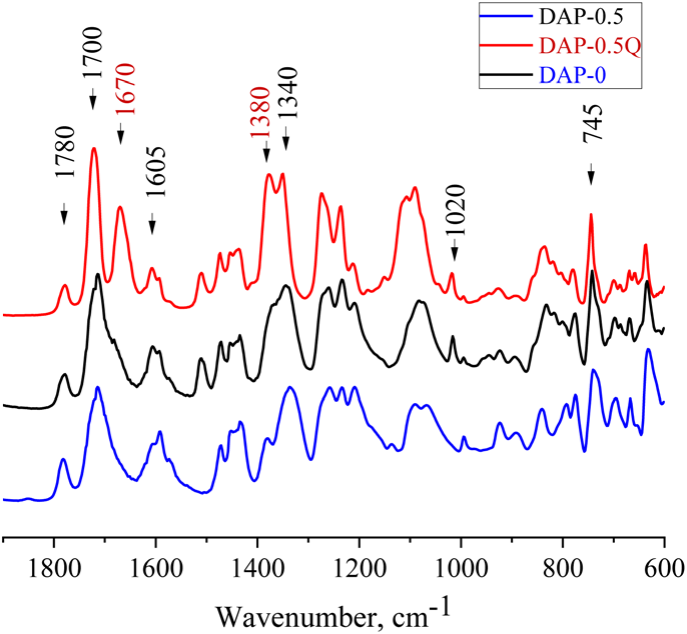
FTIR spectra of DAP-0, DAP-0.5, and DAP-0.5Q.

To prove the complete imidization of all the prepared *co*PIs, FTIR analysis was used (Fig. 1). The characteristic absorption bands of the imide group are observed at about 1700 and 1780 cm^−1^ (symmetric and asymmetric stretching of the ring carbonyl groups), the bands at 1020 and 745 cm^−1^ correspond to imide ring deformation, and 1340 cm^−1^ (stretching of the ring C–N bond), whereas the absorption band in the region of 1650 cm^−1^ corresponding to the amide bond of the corresponding PAAs is absent in the spectra of the PI and *co*PIs, which demonstrates the full imidization. With the growth of pyridine content in the *co*PI, an increase in the intensity of the bands at 1591 and 1450 cm^−1^ was observed (C=N and C–N pyridine ring stretching). In the spectra of the quaternized samples, a new peak was observed at 1670 cm^−1^, which corresponds to the C=N^+^ stretching vibration.^31,32,36^

The experimental BIS P/DAP ratio in the synthesized *co*PIs was evaluated using ^1^H NMR spectra.

**Fig. 2:**
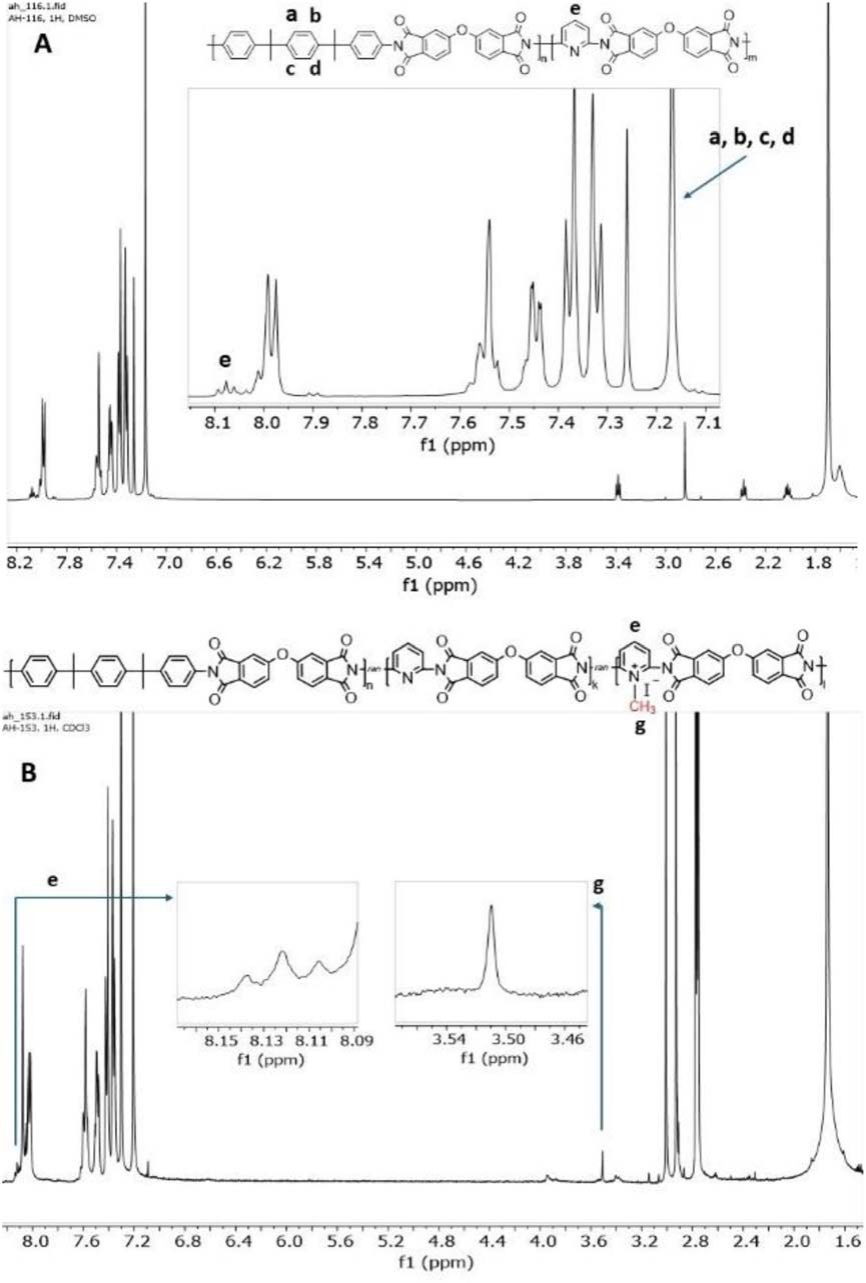
^1^H NMR spectrum of DAP-0.2 (A) and DAP-0.3Q (B).

The content of BIS P and DAP was calculated from ^1^H NMR by integrating the signal of 4 protons of BIS P *I*_(a,b,c,d)_ and the proton in the *para* position in the pyridine ring *I*_(e)_ (Fig. 2A) as follows: DAP : BIS P = *I*_(e)_/1H : *I*_(a,b,c,d)_/4H. The calculation confirmed the successful synthesis, providing products of the desired composition (Table S1†).

One of the aims of this research was to develop charged *co*PIs that could form films (membranes) from the solution of volatile organic solvent. Thus, the solubility of the obtained quaternized copolyimides (*co*PI-Qs or DAP-Qs) was investigated (see Table 1). With the increase in the pyridine content, the solubility of the samples decreases.

**Table 1:** Solubility of *co*PIs and modified *co*PIs^a^

Sample	CHCl_3_	CH_2_Cl_2_	NMP	DMF	DMSO	CH_3_CN
DAP-0.6	—	—	—	—	—	—
DAP-0.5	+	—	+	+*	—	—
DAP-0.3	+	+	+	+*	—	—
DAP-0.2	+	+	+	+*	—	—
DAP-0	+	+	—	—	—	—
DAP-0.2Q	+	+/−	+	+*	+/−	—
DAP-0.3Q	+	+/−	+	+*	+/−	+/−
DAP-0.5Q	+/−	+/−	+	+*	+/−	+/−

a “+” – Soluble, “+/−” – partially soluble, “−” – insoluble, and “+*” – soluble after heating up to 40 °C.

The *co*PIs with DAP content >50% (DAP-0.6) are brittle insoluble films. Thus, for modification, the samples with lower content of pyridine were chosen, namely, DAP-0.2, DAP-0.3, and DAP-0.5.

After quaternization, all the synthesized DAP-Qs demonstrated solubility in NMP. However, the films cast from NMP were non-transparent and fragile, containing the residual solvent even after solvent removal treatment. The DAP-Qs were also tested for solubility in chloroform, and it was shown that only DAP-Qs with the content of DAP below 30% were soluble and could form the film. In case of DAP-0.5Q, the small fragments of *co*PI-Q remained insoluble; thus, for this sample, the solution was filtered prior to casting. For these reasons, chloroform was selected as the appropriate solvent. The membranes were cast from the chloroform solution on the glass substrate and kept overnight in a chamber with chloroform vapours in order to allow them to dry slowly, thus avoiding the formation of the pores.

### Degree of quaternization and charge distribution

For samples soluble in chloroform, namely, DAP-0.2Q, DAP-0.3Q (reaction time 24 h), DAP-0.2Q2 and DAP-0.3Q2 (reaction time 48 h), the degree of quaternization (DQ) was calculated from the ^1^H NMR spectra as follows: DQ = (*I*_g_/3)/(*I*_e_ + *I*_g_/3), where e is the proton in the *para* position in the pyridine ring and g are three protons of the methyl group attached to pyridinium nitrogen, and *I*_g_/3 is the proton in the *para* position of pyridinium; thus, (*I*_e_ + *I*_g_/3) is the total amount of *para* protons both in pyridine and in pyridinium (Fig. 2B).^33^ The results are given in Table 2.

**Table 2:** The degree of quaternization of pyridine and ionic conductivity

Sample	Iodine at% theoretical	Iodine at% EDX	DQ (EDX)	DQ (NMR)	Ionic conductivity, S m^−1^
DAP-0.2Q	3.8	0.44	0.11	0.10	1.2 × 10^−3^
DAP-0.3Q	5.9	0.75	0.12	0.12	1.7 × 10^−3^
DAP-0.5Q	10.0	2.26	0.22	n/a	n/a
DAP-0.2Q2	3.8	0.59	0.15	0.15	1.8 × 10^−3^
DAP-0.3Q2	5.9	0.96	0.16	0.15	23.4 × 10^−3^

DQ was also evaluated from the data obtained by means of energy-dispersive X-ray spectroscopy (EDX). As a reference element, iodine was taken, which is absent in the initial *co*PIs and present in the *co*PI-Qs in the form of iodide ion as the counter ion of pyridinium. The theoretically calculated atomic percentage of iodine in the samples in the case of 100% conversion of pyridine to pyridinium and the iodine amount experimentally obtained *via* EDX are presented in Table 2 as well as the calculated DQ of pyridine groups.

Both methods show almost the same results. It could be seen that the DQ slightly increases with the pyridine content in *co*PIs and the reaction time. However, the DQ for all the samples is rather moderate, which can be explained by the tangled access of the quaternizing agent to the reaction centres caused by the low flexibility of the rigid pyridine-containing fragments.

The distribution of the charge (cations) in the polymer was evaluated by the elemental mapping of iodine using energy-dispersive X-ray spectroscopy (EDX).

**Fig. 3:**
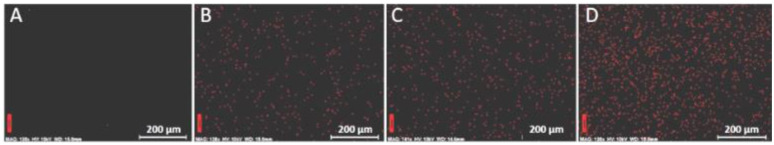
Energy dispersive X-ray spectroscopy elemental maps of iodine in DAP-0.3 (A), DAP-0.2Q (B), DAP-0.3Q (C), and DAP-0.5Q (D).

Fig. 3 demonstrates the homogeneous distribution of the iodine atoms in the bulk of modified *co*PIs, indicating uniform charge distribution throughout the polymer.

Both methods reveal that DQ grows both with the increase in the pyridine content and reaction time.

### Thermal properties of *co*PIs and modified *co*PIs

**Fig. 4:**
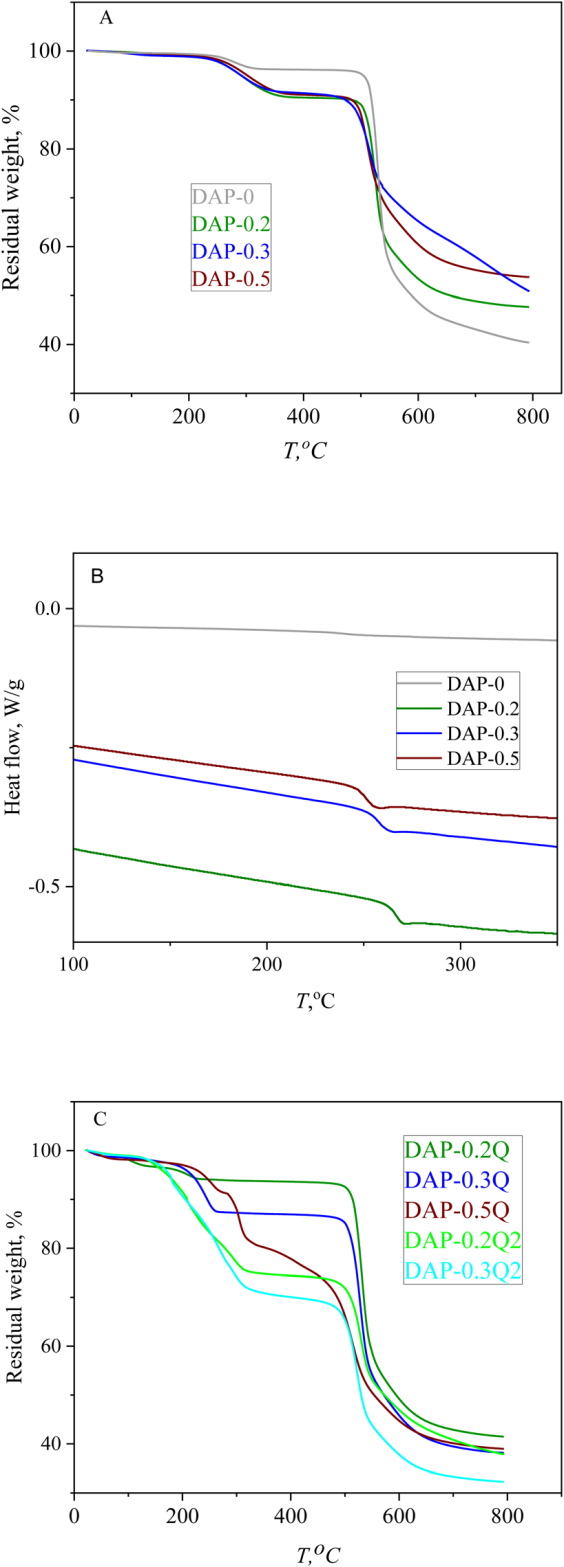
TGA curves of *co*PIs (A), DSC curves of *co*PIs (B) and TGA curves of modified *co*PIs (C).

The thermal properties of *co*PIs and modified *co*PIs were explored by means of TGA and DSC. The graphs of the thermal degradation of the *co*PIs and modified *co*PIs are given in Fig. 4.

10% loss of the weight (*T*_D10_ in Table 3) occurs at 480 °C for all the *co*PIs. The weight loss between 200 and 300 °C can be the result of the evaporation of the residual solvent (the residual NMP is present in the NMR of the *co*PIs) and/or eventually incomplete imidization.^37^

**Table 3:** Thermal properties of *co*PIs and modified *co*PIs

Sample	*T*_D10_, °C	*T*_1st_, wt%	*I*_EDX_ wt%	*T*_g_, °C
DAP-0	518	—	—	242
DAP-0.2	482	—	—	265
DAP-0.3	492	—	—	257
DAP-0.5	484	—	—	251
DAP-0.2Q	514	6	3, 9	n/a
DAP-0.3Q	245	12	7, 2	n/a
DAP-0.5Q	291	20	17, 3	n/a
DAP-0.2Q2	208	24	5, 03	n/a
DAP-0.3Q2	205	27	8, 47	n/a

Modified *co*PIs do not show noticeable weight loss up to 200 °C, then the first stage weight loss from 10 to 20% is observed in the temperature range of 200–300 °C, and the lower temperature of the weight loss can be explained by the weaker intermolecular interactions of the *co*PI-Q. The main weight loss stage for all the modified samples starts at about 500 °C and is similar to that of *co*PIs. The thermal stability of the pyridinium-containing polymers is highly dependent on the counter-ion nature, and often the earlier degradation is induced by the counter-ion at a much lower temperature than that required for backbone decomposition. For the modified *co*PIs, the first stage of weight loss at about 250 °C is similar for the DAP-0.2Q sample, but with the increase in the pyridinium content, the weight loss increases respectively due to the probable loss of iodide anions.^38,39^ The weight loss at the first stage of thermal degradation correlates with the iodine content according to the EDX data (*T*_1st_ and *I*_EDX_ columns in Table 3). The modified *co*PIs do not undergo thermal degradation at 100 °C, which is important for their performance in AEM WE with working temperature of about 80 °C.

All the *co*PIs demonstrate higher *T*_g_ then DAP-0. It can be the result of the incorporation of the “rigid” pyridine fragments into the backbone. At the same time, *T*_g_ decreases for the range of *co*PIs DAP-0.2 > DAP-0.3 > DAP-0.5 with the growth of pyridine content. Considering that the thermal degradation of the modified *co*PIs starts at about 200 °C, the *T*_g_ value for the modified *co*PIs was not obtained.

### Morphology

The morphology of the *co*PI films and modified *co*PI membranes was investigated by SEM both on the surface and at the cross section.

**Fig. 5:**
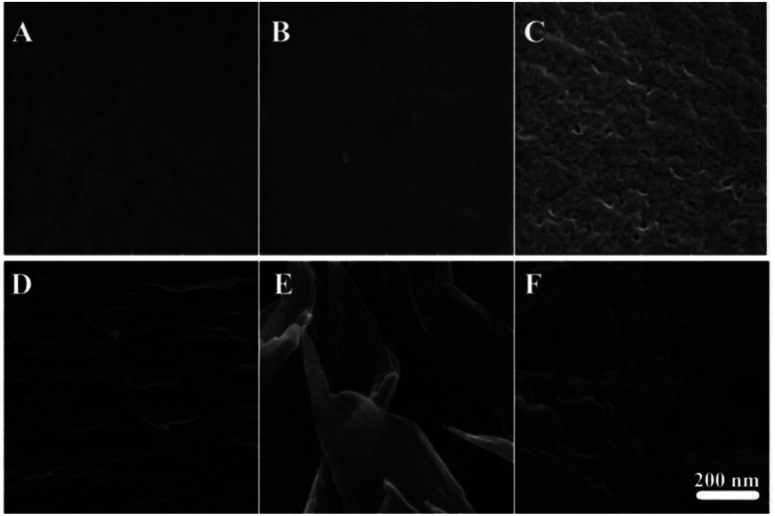
SEM images of the surface (A–C) and cross-section (D–F) of DAP-0.2Q (A and D), DAP-0.3Q (B and E), and DAP-0.5Q (C and F).

All samples are dense films. Both surface and cross section SEM images (Fig. 5) reveal the absence of pores and scratches caused by solvent evaporation. Some pore-like structures are visible for the DAP-0.5Q sample in Fig. 5C, but this is not confirmed by the cross-sectional image. This indicates that the pore-like structures are not connected and their occurrence is probably connected with solvent evaporation. From the cross-sectional images, it is also possible to see that the modified *co*PIs are structured into layers. Probably, this structure is achieved due to slow solvent removal.

### Alkaline stability and ionic conductivity

Before measuring the conductivity of the DAP-Qs, the 1 × 1 cm^2^ membrane samples were immersed into 0.1 M NaOH solution for 10 days at 24 °C. Then, the conductivity of the samples was measured and afterwards the samples were characterized by FTIR and TGA. After exposure to 0.1 M NaOH, DAP-0.5Q lost its integrity while DAP-0.2Q, DAP-0.3Q, DAP-0.2Q2 and DAP-0.3Q2 retained their shape. The ionic conductivities of DAP-0.2Q, DAP-0.3Q, DAP-0.2Q2 and DAP-0.3Q2 were 1.2 × 10^−3^, 1.7 × 10^−3^, 1.8 × 10^−3^ and 23.4 × 10^−3^ S m^−1^, respectively (see Table 2). Generally, the values of ionic conductivity are relatively low, which is in agreement with the low content of the functional groups and DQ. On the other hand, despite the low DQ, the achieved values of ionic conductivity indicate the formation of the transport channels. It can thus be expected that DQ will increase the ionic conductivity significantly.

DAP-0.3Q2 exhibits the highest ionic conductivity among the samples. This is likely due to its relatively high pyridine content and increased DQ, and as a result, the formation of more cationic centres, reaching the percolation level and thus interconnecting individual conductive domains. However, increasing the pyridine content also results in the insolubility of *co*PI, fragility of films formed from solvents like NMP, and alkaline instability, as demonstrated by DAP-0.5Q. Therefore, to further enhance the ionic conductivity, achieving higher DQs for the DAP-0.3 sample is essential. Regarding the postpolymerization modification approach used in this research to synthesize *co*PI-Q, the most straightforward method to increase DQ would be to extend the reaction time. As shown in Table 3, for DAP-0.2, extending the reaction time from 24 hours to 48 hours increases the DQ from 0.10 to 0.15. However, for DAP-0.3, the increase is less significant, rising from 0.12 to 0.15 over the same period. Thus, enhancing the DQ for DAP-0.3 requires a more complex approach and further research.

**Fig. 6:**
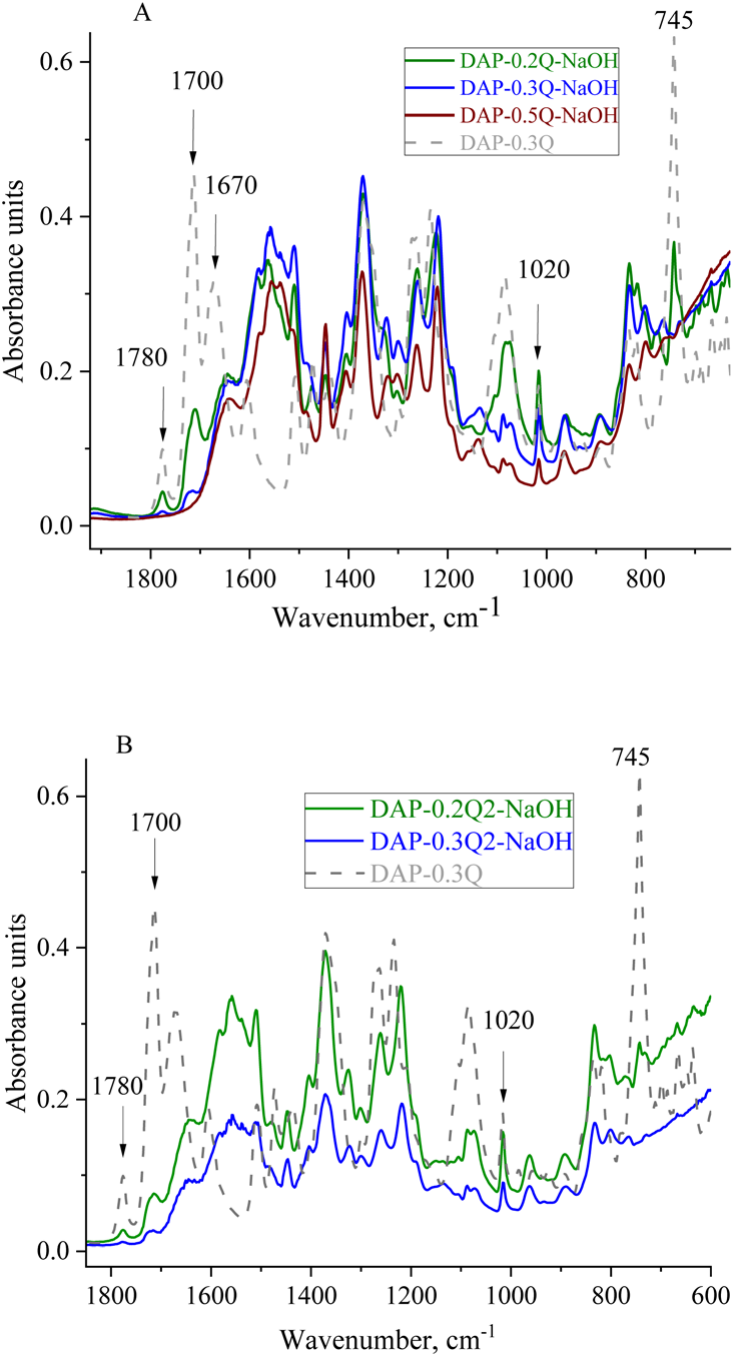
FTIR spectra of DAP-0.3Q against DAP-Qs after exposure to 0.1 M NAOH solution (A) and DAP-0.3Q against DAP-Q2s (B).

The chemical structure and thermal properties of *co*PI-Qs after alkaline treatment were investigated *via* FTIR and TGA. In the FTIR spectra (Fig. 6), changes are observed at 1700 and 1780 cm^−1^ (stretching of the ring carbonyl groups), as well as at 1020 and 745 cm^−1^, which are attributed to the imide ring. For the sample DAP-0.5Q, these bands are absent in the FTIR spectrum, which shows the degradation of the polymer backbone. DAP-0.2Q and DAP-0.3Q preserve all the bands attributed to PI. Similar behaviour is observed for DAP-0.2Q2 and DAP-0.3Q2 in Fig. 6B.

For all the samples, the band at 1670 cm^−1^ disappeared but the band at 1640 cm^−1^ appeared. This band can be observed in the spectra of all the samples and can be attributed to pyridinium against OH^−^ as the counter ion or carboxyl group formed as a result of PI backbone destruction.^40^ Regarding these possibilities, TGA of the membranes was performed.

**Fig. 7:**
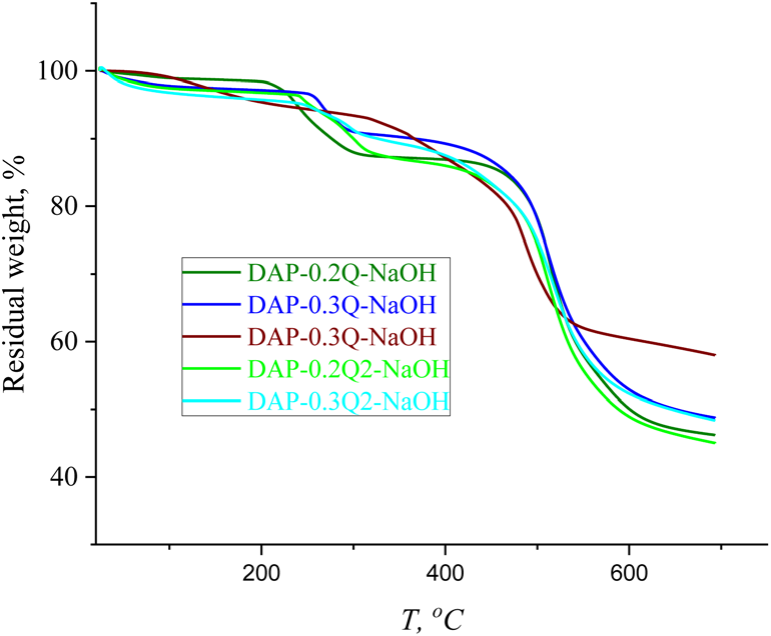
TGA curves of DAP-Qs against DAP-Qs after exposure to 0.1 M NaOH solution.

In the TGA curves in Fig. 7, there are two main stages of weight loss: the first stage can be caused by the loss of absorbed water (up to 150 °C), followed by the degradation of pyridinium hydroxide with the maximum weight loss at 250 °C,^41^ while the second stage at 460 °C is the common stage for all the PIs and *co*PI-Qs (Fig. 4C), which confirms that DAP-0.2Q, DAP-0.3Q, DAP-0.2Q2 and DAP-0.3Q2 probably did not undergo polyimide backbone degradation.

## Conclusions

Cationic *co*PIs with 2,6-protected pyridinium fragments in the main chain were synthesized by two-stage thermal imidization, followed by postpolymerization modification. The obtained *co*PIs with the content of pyridinium up to 30% were able to form dense layered membranes from chloroform solution. The obtained *co*PI-Qs showed no significant weight loss up to 200 °C. The increase in the pyridine content over 30% leads to the disintegration of the polymer backbone in alkaline medium even at low NaOH concentration. Relatively low conductivity of DAP-0.2Q, DAP-0.3Q, DAP-0.2Q2 and DAP-0.3Q2 can be the result of the low degree of substitution and low content of cations. This statement is partially confirmed by the increase in the conductivity of the DAP-0.3Q2 of about 0.234 S cm^−1^, which has the highest pyridine content and the highest conductivity value among all the studied samples. The possible way to increase the degree of substitution is to facilitate the access to the pyridine in the backbone by increasing the flexibility of the chain. This can be achieved using a pyridine-containing monomer with flexible spacers in the 2,6 positions.

## Data availability

The data supporting this article have been included as part of the ESI.† Data for this article are available at Zenodo at https://zenodo.org/records/13384465.

## Author contributions

Anastasiia Hubina – visualization conceptualization, methodology, investigation, writing – original draft, Alina Madalina Darabut – investigation, Yevheniia Lobko – investigation, Jaromir Hnat – methodology, investigation, data curation, writing – review & editing, Jan Merna – investigation, writing – review & editing, Karel Bouzek – writing – review & editing.

## Conflicts of interest

There are no conflicts to declare.

## Supplementary Material

RA-014-D4RA06443G-s001
