# Supplementary material for: Synthesis and characterization of soluble pyridinium-containing copolyimides
Source: RSC Adv. 2024 Nov 21;14(50):37278–85. doi: 10.1039/d4ra06443g (PMC11580009; doi:10.1039/d4ra06443g)
Supplement: RA-014-D4RA06443G-s001 [file RA-014-D4RA06443G-s001.pdf]

Supplementary Information (SI) for RSC Advances.
This journal is © The Royal Society of Chemistry 2024

Supplementary Materials: Synthesis and Characterization of Soluble Pyridinium Containing Copolyimides

Anastasiia Hubina, Alina Madalina Darabut, Yevheniia Lobko, Jaromir Hnat, Jan Merna, Karel Bouzek*

Dr. A. Hubina, Prof. J. Merna, Dr. J. Hnat, Prof. K. Bouzek

University of Chemistry and Technology (UCT) Prague, Prague 166 28, Czech Republic

hubina@vscht.cz

Dr. Y. Lobko, A.M. Darabut

Charles University, Prague 180 00, Czech Republic

Table S1. Monomers ratio in synthesized coPIs calculated from ^{1}H NMR spectra.

Sample	Theoretical ODPA:BIS P:DAP	NMR calculated
DAP-0.5	1:0.5:0.5	1:0.53:0.47
DAP-0.3	1:0.7:0.3	1:0.7:0.3
DAP-0.2	1:0.8:0.2	1:0.78:0.22

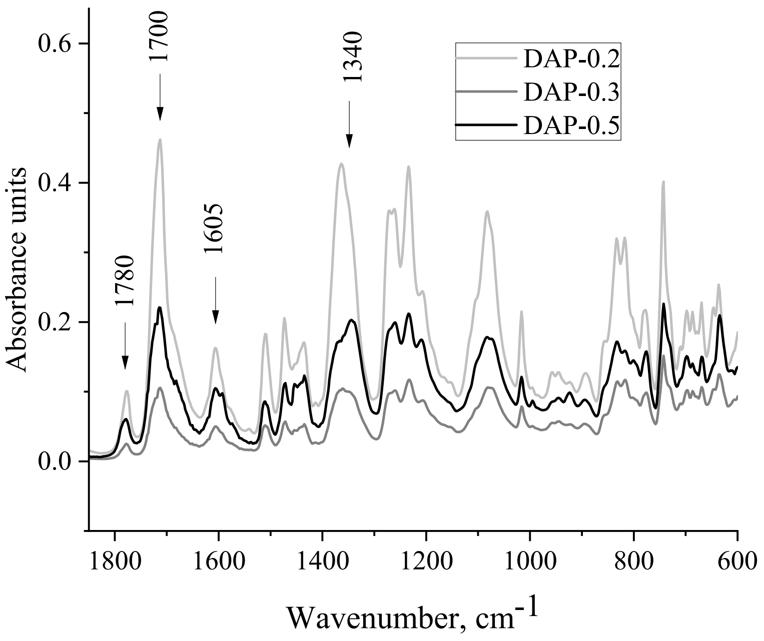

Figure S1. FTIR spectra of coPIs

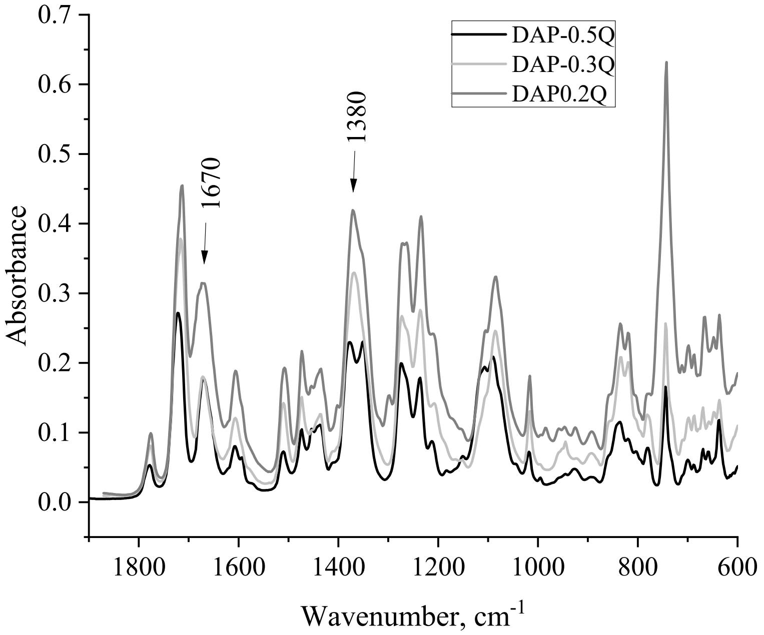

Figure S2. FTIR spectra of coPI-Qs

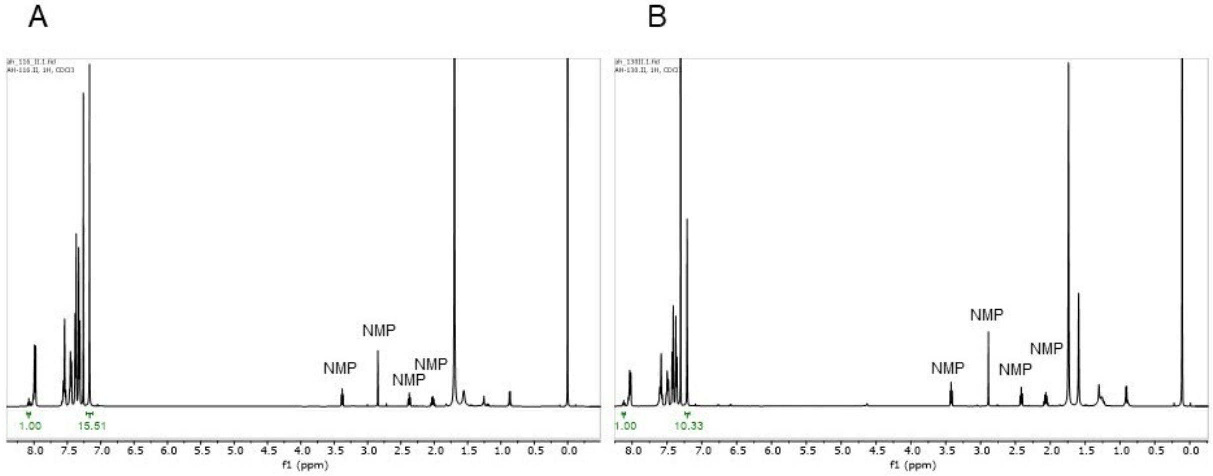

Figure S3. NMR spectra of coPIs: DAP-0.2 (A), DAP-0.3 (B)

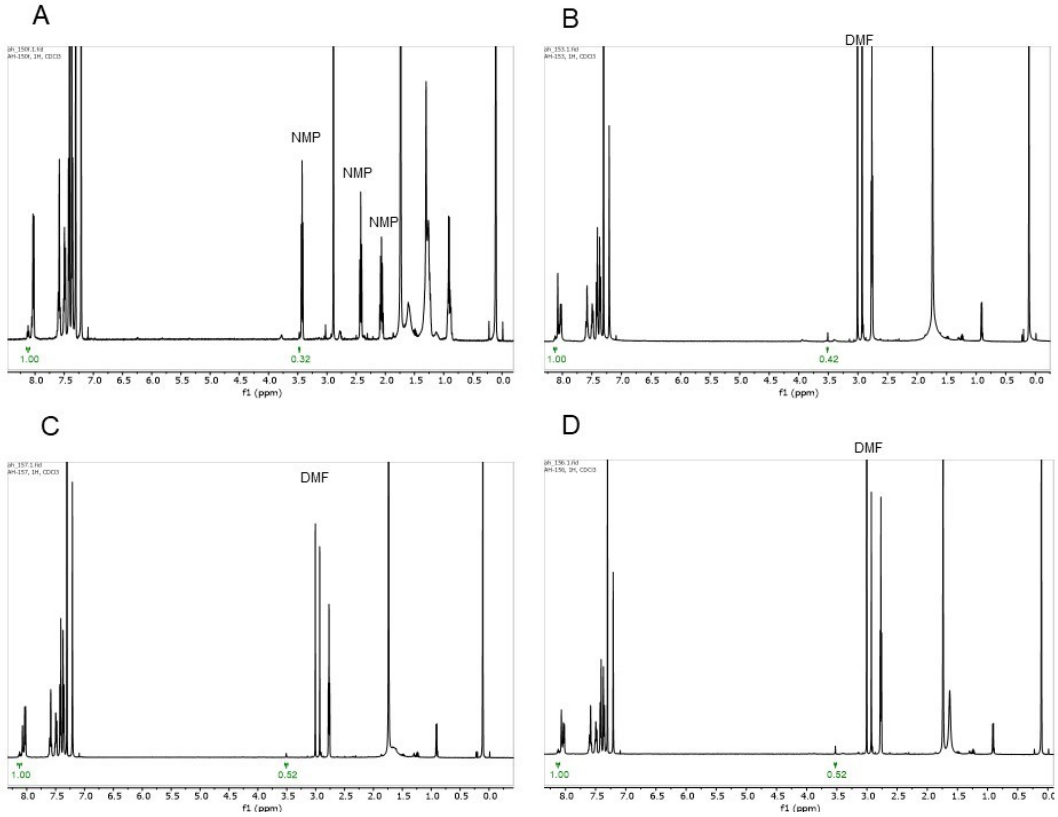

Figure S4. NMR spectra of coPI-Qs: DAP-0.2Q (A), DAP-0.3Q (B), DAP-0.2Q2-(C), DAP-0.3Q2 (D)

SEC Measurements

Molar mass and its distribution was determined using Waters SEC-RI chromatograph (Waters 515 pump, Waters 2414 refractive index detector, ECOM 4 channel degasser). Separation of polymers (~2 mg/ml solution in chloroform) was done at 25°C on Phenogel 5 μm linear column (7.8x300 mm) using chloroform (HPLC grade, stabilized by amylene) as mobile phase (1 ml/min). Evaluation of data was done in Clarity 6.0 GPC software using conventional calibration based on narrow polystyrene standards (580 – 1233000 g/mol) providing apparent molar mass values (polystyrene equivalents).

Table S2. Molar mass characteristics of PI and *co*PIs

Sample	M_n, g/mol	M_w, g/mol	D
DAP-0.5	22 100	522 000	23.6
DAP-0.3	10 900	32 700	3.0
DAP-0.2	45 100	93 400	2.1
DAP-0	38 900	89 600	2.3
